# Proton-decoupled carbon magnetic resonance spectroscopy in human calf muscles at 7 T using a multi-channel radiofrequency coil

**DOI:** 10.1038/s41598-018-24423-x

**Published:** 2018-04-18

**Authors:** Sigrun Goluch, Roberta Frass-Kriegl, Martin Meyerspeer, Michael Pichler, Jürgen Sieg, Martin Gajdošík, Martin Krššák, Elmar Laistler

**Affiliations:** 1 Division of Endocrinology and Metabolism, Department of Medicine III, Medical University of Vienna, Währinger Gürtel 18-20, 1090 Vienna, Austria; 2 Division MR Physics, Center for Medical Physics and Biomedical Engineering, Medical University of Vienna, Währinger Gürtel 18-20, 1090 Vienna, Austria; 3 Christian Doppler Laboratory for Clinical Molecular MR Imaging, Department of Biomedical Imaging and Image Guided Therapy, Medical University of Vienna, Währinger Gürtel 18-20, 1090 Vienna, Austria

## Abstract

^13^C magnetic resonance spectroscopy is a viable, non-invasive method to study cell metabolism in skeletal muscles. However, MR sensitivity of ^13^C is inherently low, which can be overcome by applying a higher static magnetic field strength together with radiofrequency coil arrays instead of single loop coils or large volume coils, and ^1^H decoupling, which leads to a simplified spectral pattern. ^1^H-decoupled ^13^C-MRS requires RF coils which support both, ^1^H and ^13^C, Larmor frequencies with sufficient electromagnetic isolation between the pathways of the two frequencies. We present the development, evaluation, and first *in vivo* measurement with a 7 T 3-channel ^13^C and 4-channel ^1^H transceiver array optimized for ^1^H-decoupled ^13^C-MRS in the posterior human calf muscles. To ensure minimal cross-coupling between ^13^C and ^1^H arrays, several strategies were combined: mutual magnetic flux was minimized by coil geometry, two LCC traps were inserted into each ^13^C element, and band-pass and low-pass filters were integrated along the signal pathways. The developed coil array was successfully tested in phantom and *in vivo* MR experiments, showing a simplified spectral pattern and increase in signal-to-noise ratio of approximately a factor 2 between non-decoupled and ^1^H-decoupled spectra in a glucose phantom and the human calf muscle.

## Introduction

Magnetic resonance spectroscopy (MRS) is a viable, non-invasive method for the assessment of cell metabolism in skeletal muscle^1,2^; in particular, ^13^C-MRS can be applied for the investigation of glycogen, triglycerides, and different intermediates of glucose metabolism. Turnover of glycogen in human muscle can be quantified from the intensity of the C-1 peak at 100.5 ppm^3–5^. Additional information can be gained by using an exercise challenge to uncover the dynamics of glycogen stores, or to study glucose/lipid oxidation in different pathologies^6–9^. ^13^C-MRS can also be used to investigate aspects of insulin resistance^10^ and type 2 diabetes^11^; specifically, the relation between muscle lipid levels and insulin resistance has been studied^12^, which is a predictor for the onset of type 2 diabetes.

However, as many NMR-detectable nuclei other than ^1^H, ^13^C signal detection suffers from intrinsically low sensitivity due to lower gyromagnetic ratio and low natural abundance of the ^13^C isotope. Experimental signal-to-noise ratio is further decreased by splitting of the resonance lines due to the ^1^H-^13^C hetero-nuclear J-coupling^13^. J-coupling can be mitigated by transmitting RF power at the proton frequency during ^13^C reception, a process called proton decoupling, which leads to a simplified spectral pattern and therefore, enhances the sensitivity of ^13^C MR measurement. In order to enable proton decoupling, the employed RF coil has to be capable of receiving the naturally low ^13^C signal while transmitting strong RF pulses at the ^1^H frequency simultaneously. This introduces additional requirements on electromagnetic isolation between ^1^H and ^13^C pathways in the RF coil and its interface^14^, approximately 100 dB of isolation is required. This can be achieved by careful coil design, as well as RF filters for additional isolation and to prevent noise injection from the RF power amplifiers^15^.

Additional sensitivity gain can be accomplished by moving to higher static magnetic field strengths (B_0_), which, in turn, results in further increase of RF power deposition since the specific absorption rate (SAR) increases with higher static field strength^16^. In order to comply with the safety guidelines issued by the International Electrotechnical Commission (IEC)^17^ or the U.S. Food and Drug Administration (FDA)^18^, the increased SAR might pose some limitation to *in vivo* ^1^H decoupled ^13^C NMR experiments. Further, the high sensitivity requirements in ^13^C NMR results in a preferred application of surface coils, due to their high SNR efficiency compared to volume coils. The lack of a broad field of view (FOV) and the inherent B_1_ inhomogeneity can be alleviated by the application of surface coil arrays^19^.

Up to now, only few RF coil arrays for ^1^H-decoupled ^13^C MRS have been presented. This is related to the increased complexity of measures for efficient isolation of ^1^H and ^13^C arrays. An often used RF coil design which fulfils the above mentioned requirements is a combination of a quadrature ^1^H coil with a linear ^13^C element^20,21^. The decoupling of the coils is achieved by geometrically arrangement, which on the other hand presents a disadvantage of this design: the elements cannot be positioned freely, and the number of elements, and thus the achievable FOV, is limited. To overcome this obstacle, parallel LC or LCC trap circuits, previously used to double tune a single loop coil^22^, have been introduced as alternative decoupling method between the ^1^H and ^13^C part^23^. These traps suppress current at the higher frequency when inserted in the lower frequency coil, and, in principle, enable free positioning of the elements. This method has been successfully applied in a double-quadrature coil for calf muscle studies^24^, and in initial work on a 4-channel ^1^H, and 4-channel ^13^C array for ^13^C-MRS in the brain^25^.

The goal of this work is to design, build, and evaluate a novel RF coil array for {^1^H}-^13^C MR metabolic measurements of the human calf muscle. Therefore, we aim for excellent ^13^C sensitivity and SAR-efficient ^1^H transmission, with sufficient isolation between ^13^C and ^1^H parts. The performance of the developed coil is optimized using electromagnetic simulations, and evaluated on the bench, as well as in phantom experiments. Finally, the feasibility of ^1^H-decoupled ^13^C-MRS with the coil *in vivo* is investigated.

## Results

### Coil design

**Figure 1:**
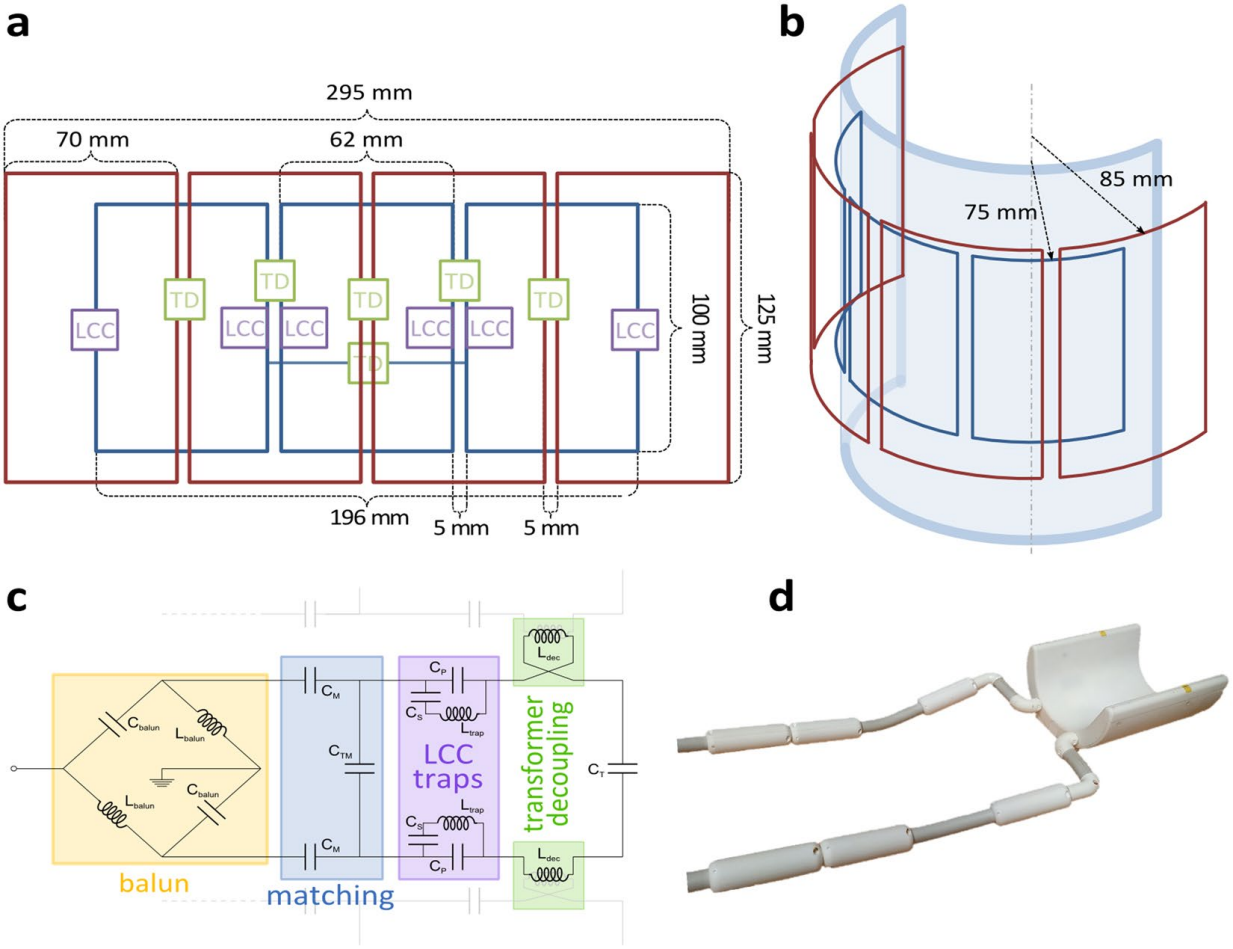
Coil design. (**a**) Unrolled view of the design. Red and blue rectangles represent the 4 channel ^1^H and 3 channel ^13^C arrays, respectively. Locations of decoupling inductances are marked by green boxes labelled TD, whereas locations of the LCC traps are marked by violet LCC-boxes. (**b**) 3D view of the RF coil with respective radii. (**c**) Equivalent circuit of the central ^13^C element, incorporating balun and matching network, coil capacitors, decoupling inductances, and LCC trap circuits. Neighbouring coil elements are indicated in light grey. (**d**) Final coil set-up with 3D printed housing, and cable strands.

Design considerations for the ^1^H and ^13^C transceiver coil arrays resulted in the geometry depicted in Fig. 1. The nested, half-cylindrical coil consists of a 4-channel ^1^H array atop of a 3-channel ^13^C array, designed for optimal coverage of the Gastrocnemius muscles and sufficient penetration depth to enable measurements in the Soleus muscle.

### Electromagnetic field simulations

**Figure 2:**
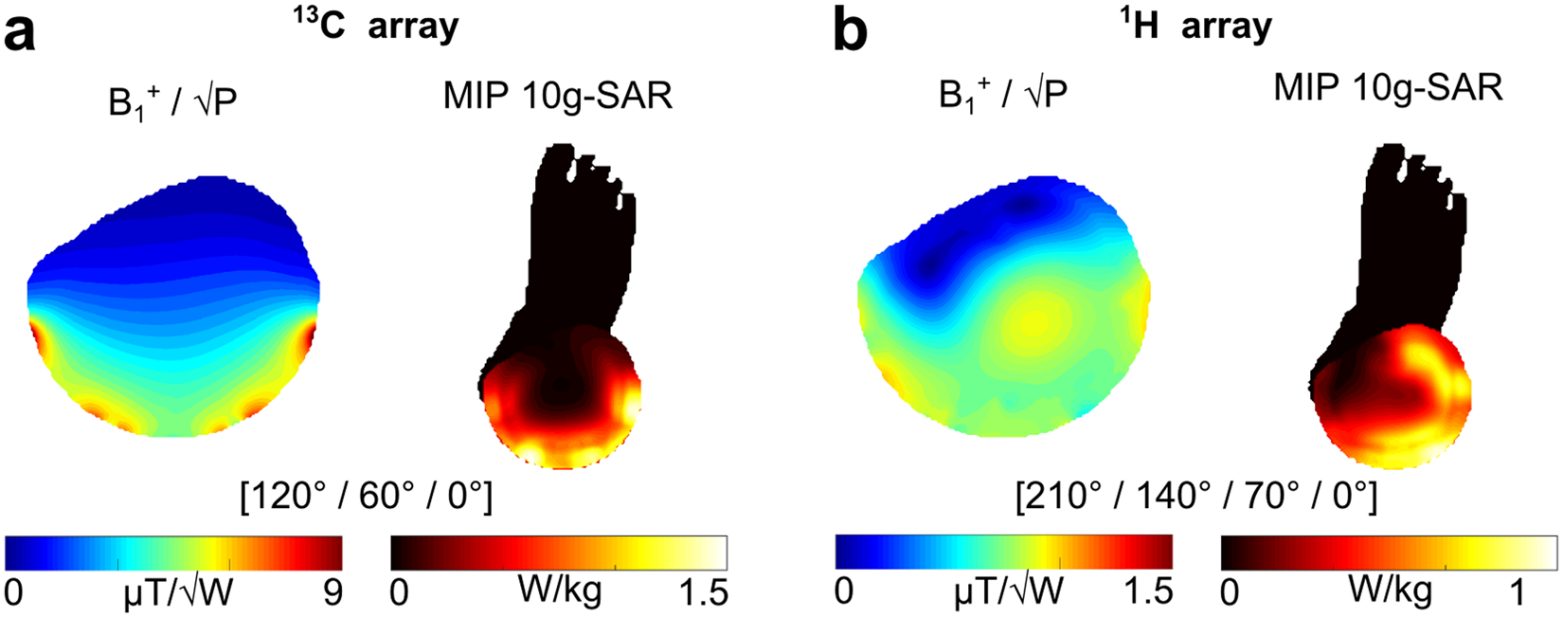
Simulation results. Transmit efficiency maps and maximum intensity projections (MIPs) of 10g-averaged SAR are shown for the (**a**) ^13^C and (**b**) the ^1^H array, using the optimum phase combination, indicated below the figures, as determined by electromagnetic simulation.

Static B_1_^+^ shimming maximizing SAR efficiency and transmit efficiency yielded phase combinations of [120°/60°/0°] for the ^13^C array and [210°/140°/70°/0°] for the ^1^H, respectively. Resulting simulated B_1_^+^ maps are shown in Fig. 2. To ensure patient safety, with the derived optimal phase setting, the maximum 10 g-averaged SAR normalized to the input power max(SAR_10g_)/P_in_, was determined as 1.0 kg^−1^ for the ^1^H array and 1.5 kg^−1^ for the ^13^C part, respectively. The simulation results were validated by MR thermometry experiments using the proton resonance frequency method^26^ and additional fibre-optic temperature sensors.

### Bench characterization

The measured full scattering (S)-parameter matrices for human calf loading, averaged over four subjects demonstrate efficient impedance matching and mutual decoupling within each of the two arrays (see Table 1).

**Table 1:** *In vivo* coupling and matching, S-parameters (dB) averaged over four subjects (2 females, 2 males).

frequency	^13^C array @ 74.7 MHz			^1^H array @ 297.2 MHz			
channel	ch 1	ch 2	ch 3	ch 1	ch 2	ch 3	ch 4
ch 1	−19.6	−19.9	−17.2	−17.3	−15.2	−17.9	−16.1
ch 2	−19.9	−17.8	−21.2	−15.2	−24.0	−17.3	−15.3
ch 3	−17.2	−21.2	−19.6	−17.9	−17.3	−18.7	−14.3
ch 4	—	—	—	−16.1	−15.3	−14.3	−24

The averaged unloaded to loaded Q-factor ratio (Q_u_/Q_l_) for the human loading condition was 3.9 for ^13^C and 3.6 for ^1^H loops, indicating high coil efficiency in the sample noise dominated regime.

### Electromagnetic isolation between ^1^H and ^13^C signal pathways

S-parameter measurements yield cross-coupling below −27.7 dB for human calf loading at the two operating frequencies (see Table 2). This isolation can be attributed to the coil geometry with a relative shift of a half element width between the two arrays and the inserted LCC trap circuits, as S-parameters were measured directly at the coil ports, i.e. without cables and interface box. Filters built in the interface box added another −70 dB of isolation, resulting in approximately −100 dB of isolation between the frequencies. The combination of all applied measures to prevent cross-coupling between ^13^C and ^1^H arrays resulted in complete absence of spikes or other interference from the ^1^H-decoupling signal during phantom and *in vivo* MRS acquisition.

**Table 2:** *In vivo* crosstalk, S-parameters (dB) between ^13^C and ^1^H arrays averaged over four subjects (2 females, 2 males).

frequency	^13^C frequency			^1^H frequency		
channel	^13^C 1	^13^C 2	^13^C 3	^13^C 1	^13^C 2	^13^C 3
^1^H 1	−34.4	−36.9	−28.9	−34.0	−33.7	−34.3
^1^H 2	−48.8	−43.9	−37.7	−53.3	−27.7	−28.8
^1^H 3	−45.7	−36.1	−41.9	−35.2	−31.7	−40.5
^1^H 4	−29.7	−59.9	−41.4	−28.7	−33.7	−42.2

### Phantom MR measurements

**Figure 3:**
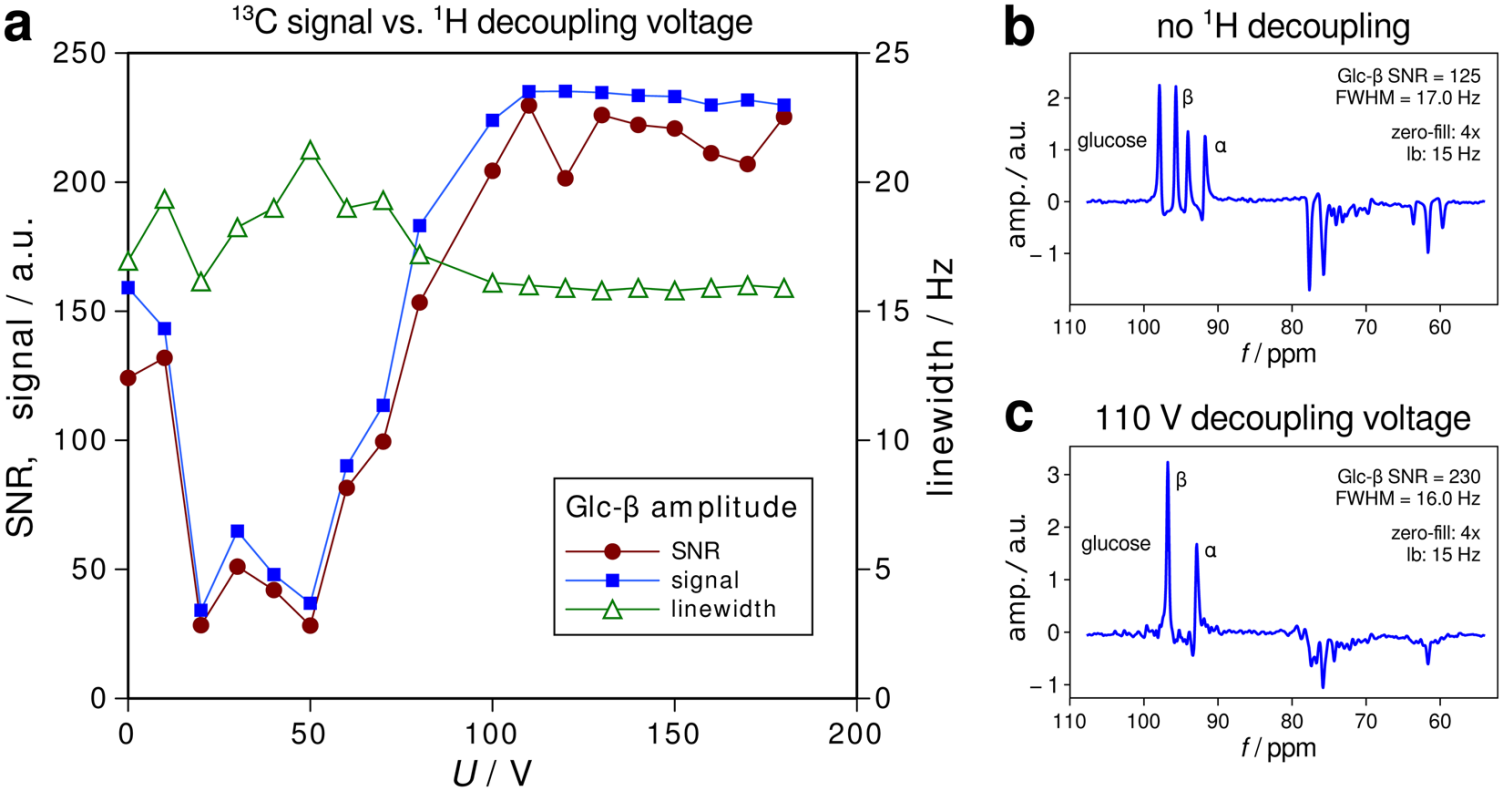
MRS results from a cylindrical phantom containing 20% natural abundance glucose solution. Spectra were acquired with 8 averages, and the voltage of the WALTZ-16 scheme, consisting of 180° block pulses with a duration of 1.5 ms, was increased from 0 to 180 V in steps of 10 V. Decoupling starts to become effective at ca. 70–80 V and reaches optimum performance at 100 V decoupling voltage and above.

^13^C-MRS experiments on a glucose phantom showed that ^1^H decoupling can be successfully performed with the developed coil, and that an SNR enhancement by approximately a factor of 2 can be achieved in comparison to the non-^1^H-decoupled case. Figure 3a shows the dependence of the SNR on the ^1^H decoupling voltage, indicating a plateau-behaviour at approximately twice the initial SNR value for voltages above 60 V. Figure 3b,c show ^13^C spectra without and with ^1^H decoupling.

### *In-vivo* MR measurements

**Figure 4:**
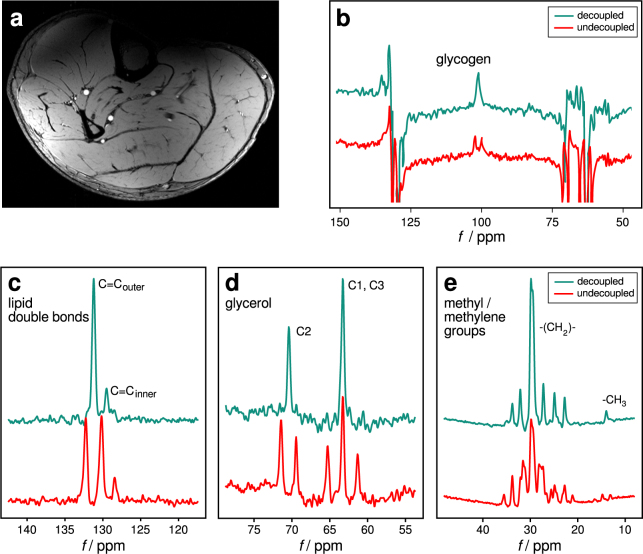
*In vivo* MR results. (**a**) Transversal 2D GRE ^1^H image of the human calf. (**b**–**e**) ^13^C spectra of human calf muscle without (red) and with (green) WALTZ-16 proton decoupling. The decoupling of all resonance lines can be clearly observed. SW = 10 kHz and 4x zero filling were consistently used. (**b**) Glycogen: TR = 1 s, 256 avgs, 512 pts, 20 Hz apodisation; (**c**–**e**) TR = 4.5 s, 16 avgs, 10 Hz apodisation; (**c**) fatty acid double bonds, 1024 pts; (**d**) glycerol, 512 pts; (**e**) methylene/methyl group of fatty acid spectra of subcutaneous adipose tissue, 512 pts.

A typical high-resolution GRE image of a volunteer’s calf (Fig. 4a) demonstrates adequate coverage of the posterior calf muscles with the ^1^H array.

**Figure 5:**
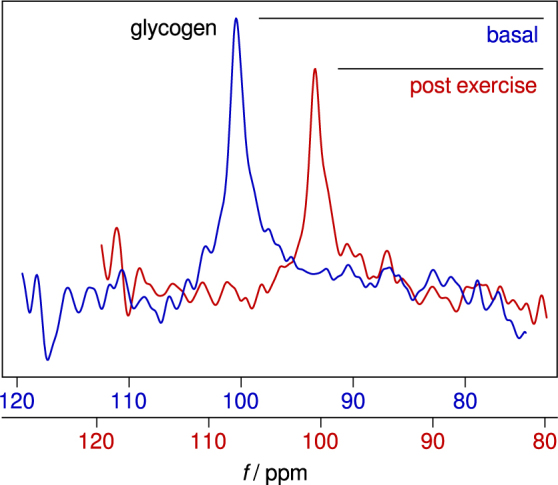
Effect of toe-raising exercise on glycogen signal. During the short toe rising exercise protocol the non-localized glycogen signal decreased by 27%.

In Fig. 4b–e, *in-vivo* ^13^C spectra without and with ^1^H decoupling are shown, demonstrating the feasibility of *in vivo* ^1^H decoupling ^13^C-MRS experiments with the developed coil. Acquired ^13^C spectra of the human calf include ^13^C resonances of natural abundance of glycogen C_1_ (100.5 ppm, Fig. 4b), lipids (30 ppm, Fig. 4e and 130 ppm, Fig. 4c), and glycerol (63 ppm and 72 ppm, Fig. 4d). For the glycogen measurement the signal increased by a factor of 1.76 ± 0.16, and the signal-to-noise ratio by factor of 1.61 ± 0.32 (both n = 5). During a short toe rising exercise protocol the non-localized glycogen signal decreased by 27%, as shown in Fig. 5.

## Discussion

In this work, a 4-channel ^1^H and 3-channel ^13^C coil array for ^13^C-MRS studies employing ^1^H-decoupling has been developed and tested.

A major criterion for the proper functionality of this coil was the electromagnetic isolation between ^1^H and ^13^C parts. This was achieved by a combination of the following measures: The number and geometric arrangement of the array elements was chosen in a way to minimize the mutual magnetic flux, and therefore the cross-coupling. Two LCC traps were inserted into each ^13^C element in order to prevent current flow at the ^1^H frequency in these elements. The cables used to connect the two arrays to the interface box were routed along separate pathways, and the interface components were arranged with a distance of >21 cm from each other. ^1^H band-pass and ^13^C low-pass filters were implemented in the interface box and in the receive path of the MR scanner in order to prevent interference effects.

The performance of the developed coil was optimized via full wave 3D electromagnetic simulations in terms of static B_1_^+^ shimming, and evaluated on the bench and in phantom MRS experiments. Finally, the feasibility of ^1^H-decoupled ^13^C-MRS with the coil *in vivo* and its value for time resolved experiments in physiology was successfully demonstrated.

Potential applications include the measurement of glucose, glycogen and lipid metabolism^6–11^ with lipid profiling skeletal muscle and subcutaneous adipose tissue of lower extremities^27^. Excitation of the full ^13^C spectral bandwidth and broadband decoupling of the full ^1^H spectral bandwidth will not be possible in a single acquisition. However, the coil is well applicable for *in vivo* studies since the different relaxation properties of glycogen and lipids favour separate tailored acquisitions of different chemical moieties of glycogen or the respective parts of the fatty acid spectrum. In interleaved experiments, information about carbohydrate and lipid metabolism of skeletal muscle^6,9,28^ can be combined. The experimental set-up of the coil with an ergometer is possible allowing for online monitoring of glycogen metabolism during an exercise challenge.

Even though the coil is tailored for direct ^13^C experiments with ^1^H decoupling it could potentially also be used for indirect ^13^C MRS, i.e. heteronuclear single-quantum coherence experiments. Due to the larger coverage and better homogeneity of the ^1^H B_1_^+^ field of our coil, the chemical shift artifact for STEAM localization in adipose tissue would increase as compared to de Graaf *et al*.^29^, but would still be within the acceptable range.

The RF coil is also applicable on the thigh, for which a good part of the literature on invasive studies (biopsies) from vastus lateralis exists. The presented device can be used as a versatile tool for applications in integrative physiology and sports sciences.

## Methods

### Coil design

Experience from previous work on 7 T calf coil design^30^ led to a half-cylindrical shape with an open diameter of 140 mm implemented as a 3D-printed, biocompatible housing (Fig. 1d), which provides mechanical stability as well as electrical and thermal insulation for the patient. The inner part of the housing conformed to the calf has a thickness of 5 mm.

Due to the intended SAR-demanding decoupling pulses at the ^1^H frequency^31^, the increase of SAR with frequency^16^, and the rapid drop-off in E-field (and, thus, SAR) with distance from the coil, the ^1^H array should be placed further away from the sample than the ^13^C array. Additionally, placing the ^13^C array immediately on the coil former increases the achievable sensitivity, resulting in a radius of 75 mm. The ^1^H array was placed 10 mm further away in radial direction resulting in a radius of 85 mm.

In view of separating ^13^C and ^1^H signals as efficiently as possible, the mutual magnetic flux should be minimized. Due to symmetry, this is achieved by geometrically choosing *n* ^1^H-elements and *n* − 1 ^13^C-elements, and shifting the arrays by a half element width. The optimal coil size^32^ equals roughly the target depth of the coil, which was set to 70 mm. Since the half circumference of a circle of radius 85 mm (=267 mm) divided by 70 mm is approximately four, this was chosen as the number of elements *n* = 4 for the ^1^H array, and consequently yielding *n* − 1 = 3 elements for the ^13^C array. Figure 1a,b show the final resulting geometry. The coil elements were manually formed from 2 mm diameter copper wire.

Nearest neighbours within each array were decoupled using counter-wound inductors in series with the coil elements (transformer decoupling, TD, Fig. 1c)^33^. To reduce coupling between next-nearest neighbours in the ^13^C array, additional transformer decoupling was introduced between the two lateral elements. TD inductors were oriented perpendicularly to the main coil wire direction to minimize interference with the B_1_ field of the coil.

Additional isolation was provided by inserting two LCC traps^23,34^ per ^13^C channel, consisting of a series inductor and capacitor in parallel with a second capacitor (Fig. 1a,c). In comparison to classic LC traps, i.e. parallel inductor and capacitor, LCC traps provide an additional degree of freedom for trap tuning, and achieve lower insertion loss at the lower frequency, where they act as a capacitor, and higher blocking impedance at the higher frequency. Trap inductors were wound in toroidal form to avoid interaction with the B_1_ field of the coil.

To minimize common mode currents, balun networks, i.e. balanced to unbalanced signal converters, were inserted between the matching circuits and the coaxial cables (Fig. 1c).

All bench measurements were conducted with a network analyser (NWA, E5071C and E5092A; Agilent, Santa Clara, California, USA). Full S-Parameter matrices were measured for both operating frequencies in order to evaluate the tuning, matching and decoupling performance of the arrays, including residual crosstalk between ^1^H and ^13^C elements. Differences in S-Parameters were investigated for different loading conditions (2 female, 2 male calves). Final tuning and matching capacitors were chosen in a way to provide a reasonable compromise in performance for all investigated human loading conditions. Tuning capacitors were distributed evenly in 4/6 gaps in each of the ^13^C/^1^H elements, respectively. Fixed-value ceramic chip capacitors (CPX Series, Exxelia Temex, Pessac, France) were used for all coil and interfacing components. Inductors were manually wound using copper wire with a diameter of 1 mm.

Quality factors of the unloaded and loaded ^1^H and ^13^C elements were calculated from the measured S_11_ curves.

### Cabling and Interfacing

**Figure 6:**
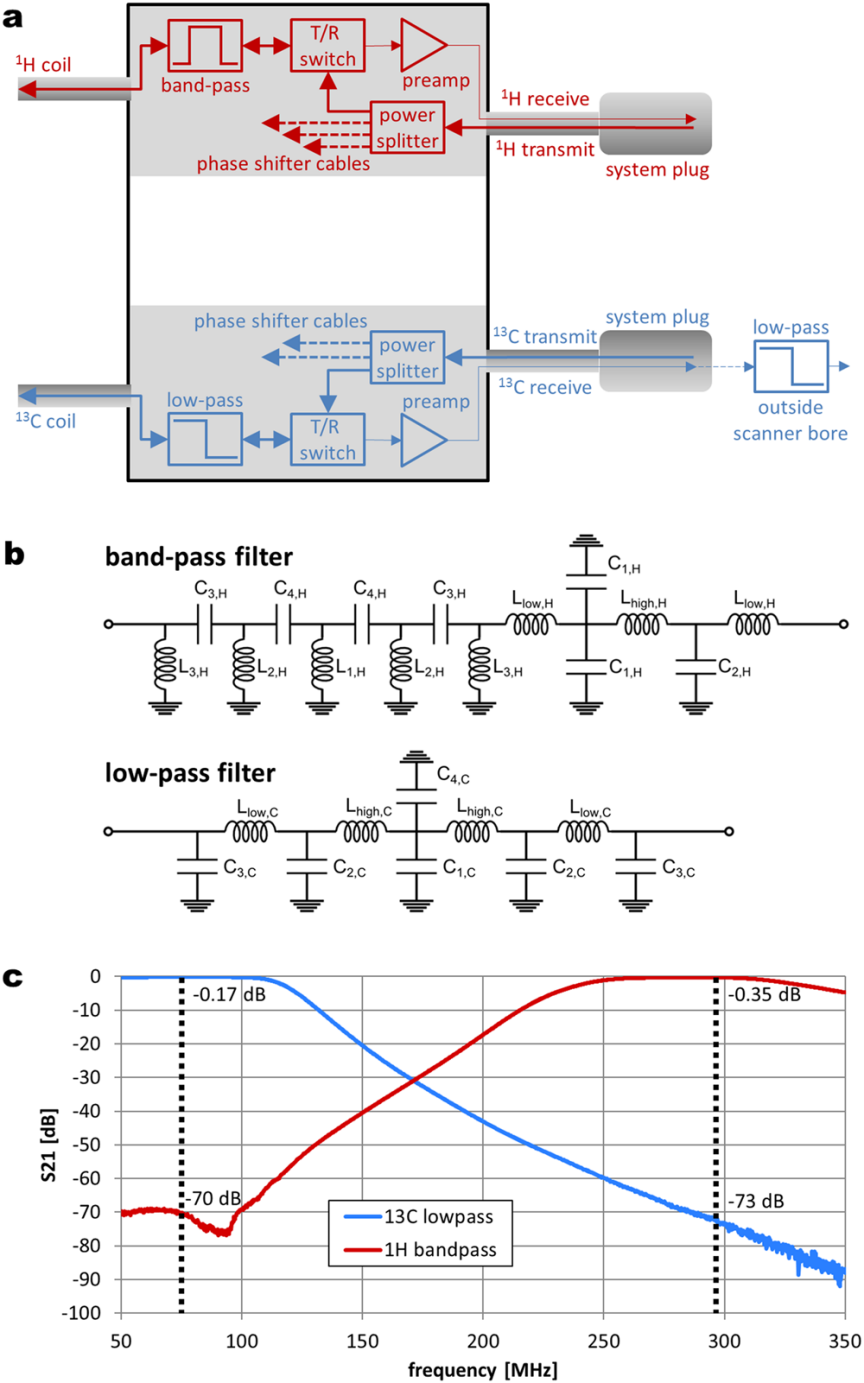
Coil-scanner interface. (**a**) Interface box incorporating Wilkinson power dividers, T/R-switches, preamplifiers, as well as band-pass and low-pass filters, respectively. The set-up is shown for one ^1^H and one ^13^C channel, only; signal paths for the other channels after the power splitters are indicated by dashed lines. For the ^13^C elements additional low-pass filters are incorporated in the receive path of the MR scanner at the far end of the scanner bore indicated by the blue box right of the system plug. (**b**) Circuit diagrams of the ^1^H band-pass (combination of Butterworth 7^th^ order low-pass and 9^th^ order high-pass filters) and ^13^C low-pass (9^th^ order Butterworth) filter, respectively. (**c**) Transmission S-parameters S_21_ for representative band-pass and low-pass filters, indicating high blocking efficiency and low insertion loss at the respective frequencies.

A separate interface box (Fig. 6a) fitting exactly to the head end of the patient bed was built. Within the interface box, components for each frequency are arranged on separate sides to minimize cross-talk. Two MR scanner system plugs, one containing all ^13^C signals (1 transmit and 3 receive coaxial lines) and one for the ^1^H signals (1 transmit and 4 receive coaxial lines), were connected to the interface box. The transmit signal was split up using 3-way and 4-way Wilkinson power dividers for ^13^C and ^1^H, respectively. Phase shifter cables of 15/60/105 cm (^13^C) and 15/28/41/54 cm (^1^H) were inserted in the transmit pathways after the power dividers. Their lengths were determined by simulation of the optimal transmit phases of the individual coil elements, as described in the following section. Transmit/receive switches were made for each coil element; preamplifiers were purchased from a third party company (Stark Contrast, Erlangen, Germany) and connected to the receive lines from the system plug. Between each transmit/receive switch and the corresponding coil element, custom-built filters were inserted to let the desired frequency pass with as low loss as possible and block the respective other frequency as efficiently as possible.

For the ^1^H lines, band-pass filters (Fig. 6b, top), a combination of Butterworth 5^th^ order low-pass and Chebyshev 9^th^ order high-pass filters was chosen. The filters were tested by measuring the transmission S-parameter S_21_ for a frequency span ranging from 50 to 350 MHz. A mean blocking efficiency of −70.4 dB@^13^C and mean insertion loss of −0.37 dB@^1^H was measured. The ^13^C low-pass filter (Fig. 6b, bottom) has a 9^th^ order Chebyshev design achieving mean blocking efficiency of −71.4 dB@^1^H, and mean insertion loss of S_21_ = −0.27 dB@^13^C. For each ^13^C channel, an additional low-pass filter with the same architecture was inserted in the receive chain of the scanner at the far end of the scanner bore (blocking −68.7 dB@^1^H, insertion loss −0.18 dB@^13^C). A typical frequency response of each of these two filter types is shown in Fig. 6c, the values for all filters are listed in Table 3.

**Table 3:** Measured transmission S-parameters (dB) for all implemented filters at the two operating frequencies, i.e. 74.7 MHz for ^13^C and 297.2 MHz for ^1^H.

filter	^1^H band-pass interface box				^13^C low-pass interface box			^13^C low-pass Rx chain		
number	1	2	3	4	1	2	3	1	2	3
S_21_@^13^C	−70.4	−67.8	−72.6	−70.1	−0.32	−0.23	−0.27	−0.18	−0.17	−0.19
S_21_@^1^H	−0.38	−0.37	−0.37	−0.35	−71.0	−71.8	−71.5	−65.5	−72.6	−71.3

To further minimize cross-talk along the signal pathways, ^1^H cables and ^13^C cables from the interface box to the coil were routed in separate strands at a distance of approximately 35 cm from each other (Fig. 1d). The cable length of 75 cm between the interface box and the coil was chosen to enable the placement of a pedal ergometer on the patient bed for dynamic exercise studies^6–8^.

In order to prevent common mode currents on the cables, floating cable traps^35^ were designed to block current at 74.7 MHz and 297.2 MHz. Three of these traps, two for ^1^H and one for ^13^C were placed alternatingly on each cable leading from the interface to the coil. Their blocking efficiency was better than −17 dB (^1^H) and −19 dB (^13^C).

### Electromagnetic Simulations

For optimisation of the coil’s transmit field via static B_1_^+^ shimming and safety evaluation, full wave 3D electromagnetic simulations were performed. The coil was modelled in XFdtd 7.4 (Remcom, State College, PA, USA) using 2 mm thick coil wire, modelled as a perfect conductor. Simulations were performed using the lower leg (knee to foot) of a digital human body model (Ella from the Virtual Family^36^) placed inside the coil model in a realistic positioning. Since the calves of the members of the virtual family are flattened on the bottom, the remaining air space between housing and tissue was filled with skin-tissue (^13^C: σ = 0.46 S/m, ε = 84.3, and ^1^H: σ = 0.64 S/m, ε = 50, ρ = 1070 kg/m^3^), in order to realize fully loaded conditions. All coil capacitors were replaced by 50 Ω voltage sources to enable circuit co-simulation in ADS (Keysight Technologies Inc., Santa Rosa, CA, USA)^37^. Realistic loss estimations for inductances, capacitances, and solder joints were modelled as series resistances. Post-processing of the simulation data was performed in Matlab (Mathworks, Natick, MA, USA) using a dedicated in-house toolbox (SimOpTx, Center for Medical Physics and Biomedical Engineering, Medical University of Vienna, Austria) employing the quadratic form power correlation matrix formalism^38,39^.

Static B_1_^+^ shimming was achieved for both arrays by maximizing SAR efficiency $(\bar{{B}_{1}^{+}}/\sqrt{max(SA{R}_{10g})})$ in a region of interest representing the gastrocnemius muscle. Relative transmit phases between elements were incremented in 5°/10° steps, resulting in 5184/46656 phase sets for ^13^C/^1^H, respectively.

### MR Measurements

MRI experiments were carried out on a 7 T whole-body MRI system (Magnetom 7 T MRI, Siemens Medical Solutions, Erlangen, Germany) equipped with a SC72d gradient coil with maximum gradient strength of 70 mT/m and slew rate of 200 T/m/s.

To evaluate the performance of the double tuned ^1^H/^13^C transceiver coil array, ^1^H-MRI and ^13^C-MRS experiments with a glucose gel phantom (PET cylinder, length 20 cm, diameter 13.5 cm, filled with 20% natural abundance glucose solution in a polyacrylic acid gel, with NaCl added to achieve physiologic conductivity) were performed. A non-localised FID sequence was used for ^13^C-MRS using WALTZ-16 pulses^40,41^ to achieve broadband proton decoupling during ^13^C signal acquisition. The RF power required for WALTZ-16 to decouple α- and β-glucose in the phantom was calibrated by increasing the amplitude of the ^1^H decoupling pulse from 0 V (no ^1^H decoupling) to 180 V in 10 V steps. The following protocol was used: T_R_ = 3 s, number of averages = 8, vector size = 512 points, decoupling pulse duration = 1.5 ms, total decoupling duration = 90%. Resulting ^13^C spectra were processed using in-house developed Python scripts quantifying peak amplitudes in the spectral domain. Data were zero-filled by a factor of 4 and apodised using a Gaussian filter with 15 Hz line broadening. The SNR was measured on the ^13^C resonances of glucose by measuring the ratio of the amplitude of the β-resonance peak(s) divided by the standard deviation of the noise in a region of the spectra with no signal.

All regulatory requirements for the investigational use of the device in humans were met and the study was approved by the local ethics board and conducted according to the Declaration of Helsinki.

^1^H-MRI and ^13^C-MRS were performed on six healthy volunteers (2f/4m, age range 27–48 years, BMI range: 20.7–26.7 kg.m^−2^) with the thickness of subcutaneous adipose tissue (SAT) ranging from 3.1 to 10.6 mm, after giving written informed consent. The volunteers were measured in feet first supine position.

One of the male volunteers (27 y, BMI 21.6, SAT thickness 4.7 mm) underwent the measurement for decoupling of fatty acid chains and glycerol resonances of SAT and a short exercise protocol containing 90 sec of toe raising at a frequency of 0.5 Hz challenging the gastrocnemius muscle. In this case, proton decoupled carbon MR spectra were acquired before and immediately after the exercise, with 5 min for volunteer placement, coil adjustment and shimming.

For the other five volunteers, the following protocol was measured: a high resolution 2D gradient echo image of the calf (T_R_ = 12 ms, T_E_ = 5.62 ms, 0.28 × 0.28 mm^2^ in-plane resolution, 3 mm slice thickness, 1 slice, 32 averages, T_acq_ = 2:46 min) was acquired to demonstrate the coverage and penetration depth achievable with the ^1^H array. Non-localised FID sequences were implemented for ^13^C-MRS experiments with and without ^1^H decoupling. A WALTZ-16 scheme^40^ with an amplitude of 100 V, 1.5 ms duration for the 180° pulse elements, and a total duration of 90% of the acquisition window was used to achieve broadband proton decoupling during ^13^C signal acquisition. Other sequence parameters are given in the caption of Fig. 4.

*In vivo* ^13^C spectra were also processed using in-house developed Python scripts, zero-filled and apodised using a Gaussian filter. The signal-to-noise ratio and the signal amplitude of the glycogen resonance with and without proton decoupling were calculated for performance evaluation of the coil.

### Data availability statement

The datasets generated during and/or analysed during the current study are available from the corresponding author on reasonable request.
